# A conserved protein of *Babesia microti* elicits partial protection against *Babesia* and *Plasmodium* infection

**DOI:** 10.1186/s13071-023-05825-x

**Published:** 2023-08-30

**Authors:** Yao Wang, Qianqian Zhang, Wanruo Zhang, Junhu Chen, Jianfeng Dai, Xia Zhou

**Affiliations:** 1 School of Biology and Basic Medical Sciences, Soochow University, No.199 Renai Road, Suzhou, 215123 People’s Republic of China; 2 Institutes of Biology and Medical Sciences, Jiangsu Key Laboratory of Infection and Immunity, Soochow University, No.199 Renai Road, Suzhou, 215123 People’s Republic of China; 3 National Institute of Parasitic Diseases, Chinese Center for Diseases Control and Prevention (Chinese Center for Tropical Diseases Research), Key Laboratory of Parasite and Vector Biology, National Health Commission of the People’s Republic of China (NHC), World Health Organization (WHO) Collaborating Center for Tropical Diseases, National Center for International Research on Tropical Diseases, Shanghai, 200025 China

**Keywords:** *Babesia microti*, Conserved protein, Vaccine, Bioinformatic analysis, Antigens

## Abstract

**Background:**

The protozoan parasite *Babesia microti* that causes the zoonotic disease babesiosis resides in the erythrocytes of its mammalian host during its life-cycle. No effective vaccines are currently available to prevent *Babesia microti* infections.

**Methods:**

We previously identified a highly seroactive antigen, named *Bm*8, as a *B. microti* conserved erythrocyte membrane-associated antigen, by high-throughput protein chip screening. Bioinformatic and phylogenetic analysis showed that this membrane-associated protein is conserved among apicomplexan hemoprotozoa, such as members of genera *Babesia*, *Plasmodium* and *Theileria*. We obtained the recombinant protein *Bm*8 (r*Bm*8) by prokaryotic expression and purification.

**Results:**

Immunofluorescence assays confirmed that *Bm*8 and its *Plasmodium* homolog were principally localized in the cytoplasm of the parasite. r*Bm*8 protein was specifically recognized by the sera of mice infected with *B. microti* or *P. berghei*. Also, mice immunized with *Bm*8 polypeptide had a decreased parasite burden after *B. microti* or *P. berghei* infection.

**Conclusions:**

Passive immunization with *Bm*8 antisera could protect mice against *B. microti* or *P. berghei* infection to a certain extent. These results lead us to hypothesize that the *B. microti* conserved erythrocyte membrane-associated protein *Bm*8 could serve as a novel broad-spectrum parasite vaccine candidate since it elicits a protective immune response against *Babesiosis* and *Plasmodium* infection.

**Graphical Abstract:**

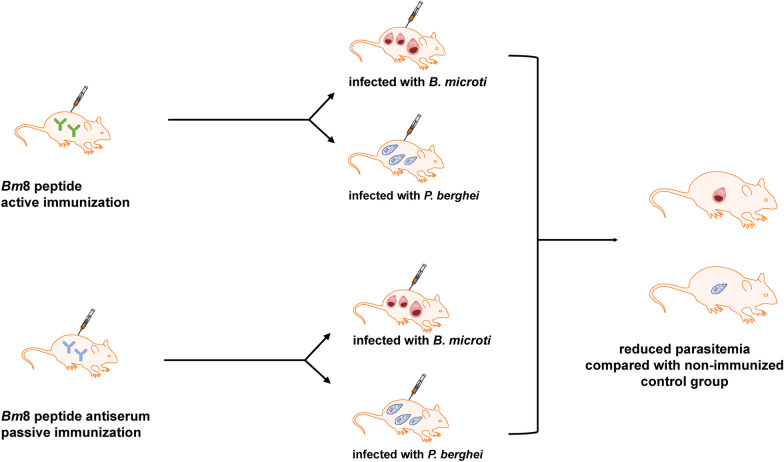

**Supplementary Information:**

The online version contains supplementary material available at 10.1186/s13071-023-05825-x.

## Introduction

Parasites of the genus *Babesia* are tick-borne intraerythrocytic protozoa belonging to the phylum Apicomplexa. Babesiosis has become an emerging public health threat and has been designated a national notifiable infectious disease in many countries [1–3]. The main *Babesia* species known to infect humans and thus function as zoonotic pathogens are *B. microti*, *B. venatorum*, *B. canis* and *B. divergens* [4, 5]. Clinical presentation of babesiosis is predominantly asymptomatic or mild symptoms, but more severe clinical symptoms are commonly found in populations of neonates or immunocompromised patients. In addition, *Babesia* infections can be life-threatening in splenectomy patients [6, 7]. *Plasmodium*, another genus of vector-borne protozoan parasites, poses an even greater threat to global health as it causes malaria, In 2021 alone, an estimated 619,000 deaths were attributed to malaria [8]. Both *Plasmodium* and *Babesia* are apicomplexan hemoprotozoa that can infect and replicate within host erythrocytes. Cytoadherence of infected red blood cells (RBCs) mediated by parasite invasion and the invasion of RBCs by parasites are two different and independent processes. As a first step in RBC invasion, the merozoites attach to the membrane of the RBCs [9, 10], a process that depends highly on interactions between the parasite and molecules present on the host cell surface [11–13]. A series of studies in animal models have shown that the protection provided by some proteins involved in cell invasion and immunity is limited; thus, to date, no vaccine is available against* B. microti* infections and further exploration is needed [14–19]. The RTS,S vaccine, which is the world's first and most highly developed malaria vaccine, demonstrated only modest efficacy against* Plasmodium falciparum*, with relatively short longevity [20].

Current vaccine development efforts are focusing on using antigenically defined immunogens, particularly those molecules interacting with or disrupting the process of parasite invasion into host RBCs [21]. In our previous proteome high-throughput screening study, we identified a number of *B. microti* proteins with high antigenicity [19, 22]. The protection and diagnostic potential of signal peptides from these secreted proteins, including protein 44, named *Bm*SP44, were evaluated [23]. In the present study, screening of proteome high-throughput chips and analysis by bioinformatics resulted in the identification of erythrocyte membrane-associated protein 8 of *B. microti* (named *Bm*8) as a conserved protein among the apicomplexan parasites, such as *Plasmodium* spp. and *Theileria* spp. After cloning and expressing the protein and synthesizing one of its peptide fragments, we evaluated the immune protection against murine *Plasmodium* infection and *Babesia* infection. This aim of this study was to identify common molecules that can be used as a reference for the prevention and control of vector-borne hemoprotozoa.

## Methods

### Mice and parasite infections

Female BALB/C mice, aged 6–8 weeks, were obtained from the Laboratory Animal Center of Soochow University and raised in a specific pathogen-free (SPF) environment. The *B. microti* Peabody strain (ATCC: PRA-99) was originally obtained from the American Type Culture Collection (ATCC, Manassas, VA, USA). Both of the parasites used in this study, namely *B. microti* and *Plasmodium berghei* ANKA, were provided by National Institute of Parasitic Diseases (NIPD), Chinese Center for Disease Control and Prevention (Beijing, China). Two BALB/c mice in each group were infected by intraperitoneal injection with parasites of *B. microti* or *P. berghei*. Blood samples from all infected animals were collected from eyelids into anticoagulant tubes containing EDTA at 5–7 days after infection (when the parasitemia was about 60%). The infected blood was then mixed with phosphate-buffered saline (PBS) in a 1:4 ratio, and each BALB/c mouse was injected intraperitoneally with 100 μl diluted blood (about 1 × 10^7^ infected parasites in RBCs [iRBCs]).

All animal experiments were conducted in accordance with the principles of ethical use of animals for medical purposes by the Ministry of Health of the People's Republic of China and approved by the Institutional Animal Care and Use Committee (IACUC) of Soochow University for the use of laboratory animals (Permit Number: ECSU-201800091).

### Sequence analysis and modeling of the crystal structure

Sequence alignment of the target *Plasmodium* erythrocyte membrane-associated gene from species of the *Babesia*,* Plasmodium* and *Theileria* genera was performed using the MEGA version X tool [24]. Phylogenetic analysis was performed with the maximum likelihood and neighbor-joining methods, and tree topologies were compared for a robust phylogeny. The sequence of *B. microti* (Accession number: XP_021338580.1), *Babesia bovis* (Accession number: XP_001610259.2), *P. berghei* (Accession number: XP_034420266.1), *Plasmodium vivax* (Accession number: SCO66108.1), *Plasmodium falciparum* (Accession number: XP_002585407.1) and *Theileria annulata* (Accession number: XP_952528.1) were blasted online [25]. *Toxoplasma gondii* (Accession number: 37589.1) was selected as an outgroup, and bootstrap values were calculated with 1000 pseudo replicates. The SWISS-MODEL (https://swissmodel.expasy.org/interactive) was used to generate the three-dimensional (3D) structure model based on the crystal structure of *Plasmodium* erythrocyte membrane-associated antigen that has been previously reported.

### Expression and purification of recombinant protein* Bm*8

Complementary DNA of *B. microti* was used to amplify the open reading frame (ORF) of *Bm*8. The full-length ORF of *Bm*8 was cloned into the pGEX-6p-2 vector using the* Bam*HI and* Xho*I restriction sites and gene-specific forward (TTCCAGGGGCCCCTGGGATCCATGCATATCAACTACAAATTAATTA) and reverse (CACGATGCGGCCGCTCGAGTTAAGCAGCATTAGGTGTGTGAT) primers. The fusion protein containing the GST tag in *Escherichia coli* BL21 was expressed using a protocol similar to that described in [23]. After cleavage of the GST tag with Procession protease, the recombinant protein *Bm*8 ®*Bm*8)was obtained and verified by sodium dodecyl sulfate–polyacrylamide gel electrophoresis (SDS-PAGE) and western blot assay.

### Antigenicity analysis of r*Bm*8 by enzyme-linked immunosorbent assay

An enzyme-linked immunosorbent assay (ELISA) was performed following standard procedures. Briefly, microtiter 96-well plates were coated with 1 μg/ml (100 μl/well) of r*Bm*8 in 0.1 M bicarbonate coating buffer (pH 9.6) and left to stand overnight at 4 °C. The plates were then washed 3 times with 200 μl of PBS plus 0.05% Tween-20 (Sigma–Aldrich, St. Louis, MO, USA), following which the wells were blocked with 1% bovine serum albumin (BSA) for 1 h at room temperature with 100 μl of blocking buffer (PBS with 0.05% Tween-20 and 5% non-fat milk). Pooled sera from mice infected with *B. microti* or *P. berghei* (6 mice in each group; sera collected 14 days post infection; 100 μl) and an equivalent amount of negative mouse sera (collected from healthy mice) were diluted with 1% BSA (1:10, 1:100, 1:1000 and 1:10,000) and incubated for 2 h. After incubation with the peroxidase-conjugated rabbit anti-mouse immunoglobulin G (IgG) antibody for 1 h, the TMB substrate (Ebioscience, California, America）for horseradish peroxidase microwell applications was added and incubated for 20 min to detect the reaction. The reaction was stopped using 100 μl of 1 M H_2_SO_4_. The optical density (OD) at 450 nm was determined with a microplate reader (model ELX800; BioTek, Winooski, VT, USA). The ELISA tests were repeated 3 times for each infected model.

### Immunofluorescent assay and confocal microscopy

Blood was collected from mice when the parasitemia was about 70%. As a first step, the blood was smeared on slides using cytospin centrifugation (Thermo Fisher Scientific, Waltham, MA, USA) and fixed with 4% paraformaldehyde-PBS for 10 min. The isolated erythrocytes on the slides were then permeabilized by 0.4% Triton for 15 min and blocked with 5% fetal bovine serum (FBS) for 30 min. Finally, the slides were incubated with anti-*Bm*8 serum diluted 1:500 overnight at 4 °C. After incubating with Alexa fluor 680 goat anti-rabbit IgG diluted 1:500 for 1 h, 0.5 ug/ml DAPI was added and stained in the dark for 10 min. Imaging was performed using the Nikon C^2+^ Confocal Microscopy system (Nikon Corp., Tokyo, Japan).

### Analysis and synthesis of *Bm*8 polypeptides and evaluation of immune protection

The antibody epitopes of *Bm*8 were predicted online by Immune Epitope Database (IEDB) analysis resources (http://tools.immuneepitope.org/bcell/). The antigenicity of *Bm*8 was analyzed, and the target polypeptides (CYDPEKSNSAEW) were selected and chemically synthesized by Sangon Biotech (Shanghai, China). The rabbit antisera were prepared by immunizing the rabbit with the synthesized polypeptides.

For active immunization, five mice in each group were immunized with *Bm*8 polypeptides (20 μg/mouse) mixed with complete Freund's adjuvant, as subcutaneous immunization after emulsifying. The control group was immunized with the mixture of Freund's adjuvant and PBS only. Booster immunization (40 μg/mouse) was carried out 2 weeks after the first immunization, followed by a second booster immunization (40 μg/mouse) 1 week thereafter. The serum was collected 1 week after the third immunization and the antibody titers measured by ELISA. If successful active immunization was confirmed by ELISA, the animals were challenged 1 week after the last booster immunization with an intraperitoneal infection of RBCs containing 1 × 10^7^ parasites of either *P. berghei* or *B. microti.*

For passive immunization, five mice in each group were immunized with *Bm*8 polypeptide antisera provided by a synthetic peptide company, Sangon Biotech (Shanghai, China) (200 μl each) or with normal rabbit sera. After 24 h, the animals were challenged with intraperitoneally infection of RBCs containing 1 × 10^7^ parasites of *P. berghei* or *B. microti*. Two days later, the mice in both groups were given one booster immunization with the same dosage as the initial immunization.

### Parasitemia and quantification of gene expression of parasites load

The concentration of parasites in peripheral blood in mice was determined by blood smear examination and real-time quantitative PCR (qRT-PCR). Blood smears were obtained from the tail tip of mice every 3 days after infection. The number of infected erythrocytes in each visual field was calculated by averaging 50 visual field counts under a microscope after Giemsa staining. The degree of infection in each mouse was expressed as the percentage of parasitemia. At the same time, RT-PCR was applied to amplify messenger RNA (mRNA) to determine parasite load. Briefly, the DNA/RNA Isolation Kit of Omega Bio-tek (Norcross, GA, USA) was used to extract mRNA from blood of infected mice. Reverse transcription was performed with 5× All-In-One RT Mastermix (abm, Shanghai, China). The following primers were used for the RT-qPCR assay: (i) *B. microti* 18S ribosomal RNA (rRNA) forward (AGCGTTTTCGAAGGTATGTTGC) and reverse (GCAGATACATCCTTACTAGGGAAA) primers; (ii) *P. berghei* 18S rRNA forward (AGCGTTTTCGAAGGTATGTTGC) and reverse (AGCAGATACATCCTTACTAGGGAAA) primers; (iii) mouse beta-actin (control gene) forward (AGAGGGAAATCGTGCGTGAC) and reverse (CAATAGTGATGACCTGGCCGT) primers. The qPCR protocol consisted of a pre-denaturation at 95 °C for 5 min, followed by denaturation for 10 s, annealing at 60 °C for 10 s and extension at 72 °C for 30 s.

### Severity of the Babesiosis and *P. berghei* infections

In addition to measuring parasitemia and the parasite load of *Babesia* or *Plasmodium*, the weight, temperature and hemoglobin (Hb) level of mice were also assessed to evaluate the severity of the mice babesiosis or *P. berghei* infections. The weight and anal temperature were monitored daily after infection with *B. microti* or *P. berghei*. To measure the concentration of hemoglobin (Hb) from different groups, 10 µl blood was diluted in 2490 µl of Drabkin's reagent (Sigma-Aldrich, St. Louis, MO, USA) in each sample and quantified at 540 nm using a biophotometer (Eppendorf, Hamburg, Germany). The absorbance and Hb concentration were counted using a commercially available Hb standard curve.

### Statistical analysis

Data were analyzed using the software GraphPad Prism 9.0 (GraphPad Software Inc., San Diego, CA, USA). Data differences between control and experimental groups were compared using an independent-sample t-test, with *P* < 0.05 as the criterion for statistical significance of the data.

## Results

### Molecular characterization and phylogenetic analysis

**Fig. 1:**
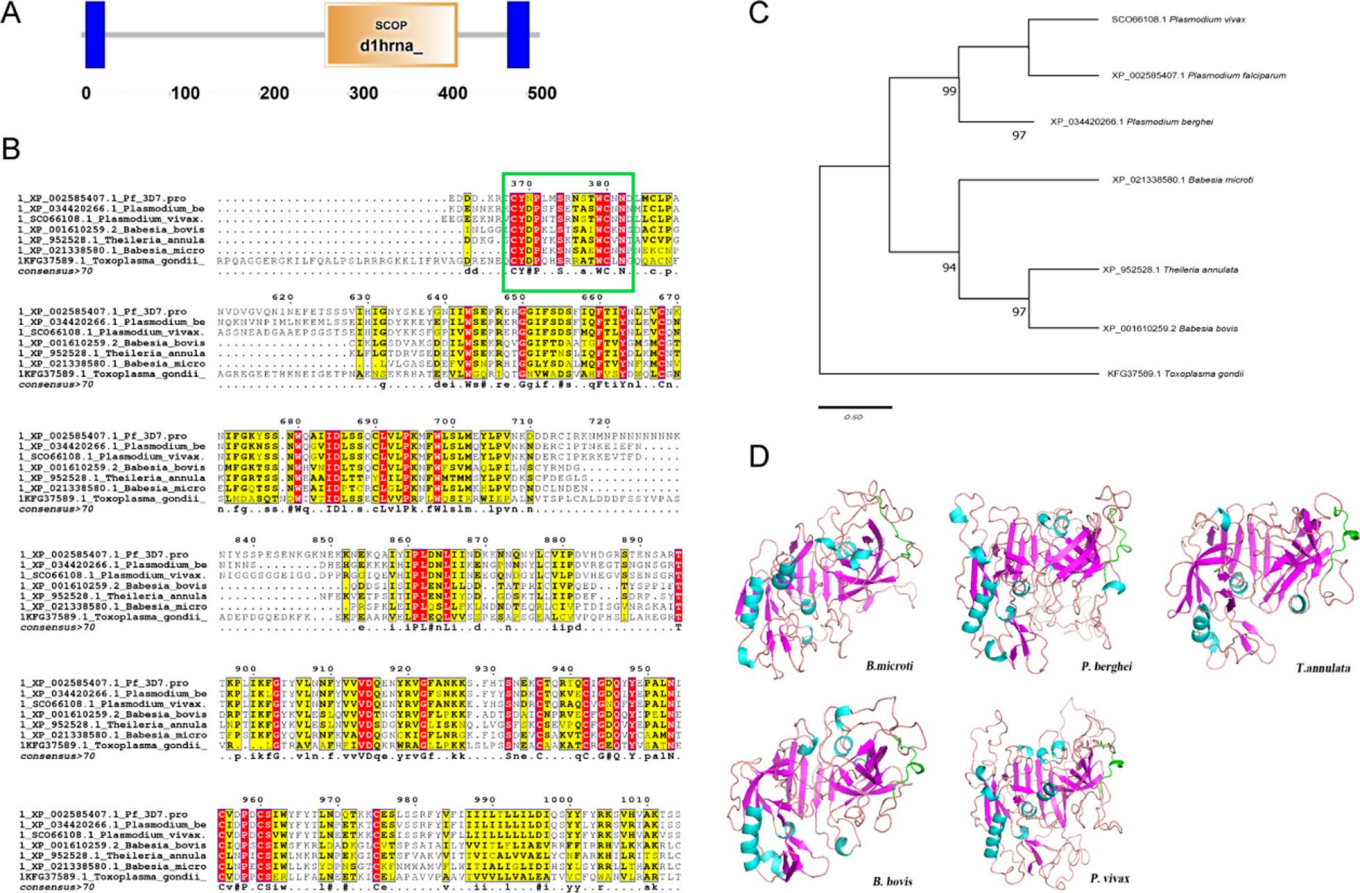
Bioinformatics analysis of the conserved membrane-associated antigen, *Bm*8. **a** Conserved domain search indicated that the protein has a homolog with a known structure at 274–423; two transmembrane regions were detected by the TMHMM Server(https://dtu.biolib.com/DeepTMHMM). Numbers from 0 to 500 represent the length of the protein sequence. The transmembrane region is shown in blue. **b** Multiple sequence alignment by ClustalW of the conserved membrane-associated sequences of *Babesia microti* with other apicomplexan protozoa (*Plasmodium berghei*, *Plasmodium falciparum*, *Plasmodium vivax*,* Babesia bovis*, *Theileria annulata* and *Toxoplasma gondii*). The green box shows the synthetic conservative polypeptide regions (i.e. all amino acids are identical); areas marked in yellow represent incompletely conserved protein regions (i.e. at least 4 of the 6 samples have identical amino acids). The green box shows the conserved polypeptides among *Babesia*, *Plasmodium, Theileria* and *Toxoplasma* with the synthesized peptides of *B. microti* selected in this study. **c** Phylogenetic tree of the sequence of the conserved membrane-associated sequences with other apicomplexan protozoa species. The scale bar represents the nucleotide substitutions per position. Branch lengths represent the amount of genetic distance change between the strains. **d** Three-dimensional structural models of erythrocyte membrane-associated conserved protein of *Bm*8 predicted by SWISS-MODEL program. The conserved membrane-associated sequences of other apicomplexan protozoa (*P. berghei*,* P. vivax*, *B. bovis,* and *T. annulata*) were predicted simultaneously. The green regions represent the high antigenic conservative polypeptides applied in this study. Bm8, *B. microti* protein 8

The coding sequence of *Bm*8 consisted of 1545 nucleotides, which was predicted to produce a protein consisting of 515 amino acid residues (Additional file 1: word file S1). The protein was estimated to have a molecular weight of 56.65 kDa and an isoelectric point of 7.91. The TMHMM Server was used to perform a conserved domain search, indicating that the protein has a homolog with a known SCOP domain structure at positions 274–423 and two transmembrane regions (referenced from https://dtu.biolib.com/DeepTMHMM), as shown in Fig. 1a. Multiple sequence alignment by ClustalW suggested that *Bm*8 shared homology with sequences of apicomplexan protozoa parasites *P. berghei* (GenBank: XP_034420266.1), *Plasmodium falciparum* (GenBank: XP002585407.1) and *Plasmodium vivax* (GenBank: SCO66108.1) (Fig. 1b). The conserved membrane-associated sequence of *Bm*8 was analyzed using a phylogenetic tree to compare it with homologs from other apicomplexan protozoa species, which revealed that *Bm*8 has a closer relationship with *Plasmodium* spp. and *Theileria* spp. The KFG37589.1.* Theileria gondii* conserved membrane-associated protein amino acid sequence was used as an out group (Fig. 1C).

### Structural analysis of the conserved membrane-associated antigen

To further confirm the conservation of this membrane-associated antigen in the apicomplexan parasites, the 3D structure of these proteins was predicted. The predicted 3D structure of the conserved membrane-associated antigen was predicted to contain seven α-helices, 22 β-strands and 279 random curls (Fig. 1d) in *Bm*8*.* Homology modeling was performed with the homology proteins from *B. microti*,* P. berghei* and *P. vivax,*, with the results showing that their predicted domain structures are well conserved. The part of the 3D structure of* Bm*8 shown in green (Fig. 1b) is the area predicted with high immunogenicity and the region of the polypeptides synthesized. Its immune protection efficacy was evaluated in this study, showing that their predicted domain structures are well conserved.

### Expression of r*Bm*8

To investigate the characteristics of the conserved membrane-associated protein *Bm*8, we generated a recombinant protein with the GST tag (approx. 24 kDa). Although the yield of rBm8 protein was relatively low in the* E. coli* expression system, the protein was successfully purified. The predicted size of r*Bm*8 was 57 kDa, which was confirmed by SDS-PAGE after purification. The western blot assay was probed with normal sera and with anti-*B. microti* mice sera infected for 2 weeks, respectively. The results of the western blot assay confirmed not only the purification of r*Bm*8, but also the specific antigenicity of r*Bm*8 (Additional file 2: Figure S1 A, B). Considering the low yield of r*Bm*8, we synthesized the epitope polypeptides to immunize mice. The predicted linear epitopes of *Bm*8 antigens were listed in Additional file 3: Figure S2 A, B, C.

### Antigenicity and subcellular localization of *Bm*8

**Fig. 2:**
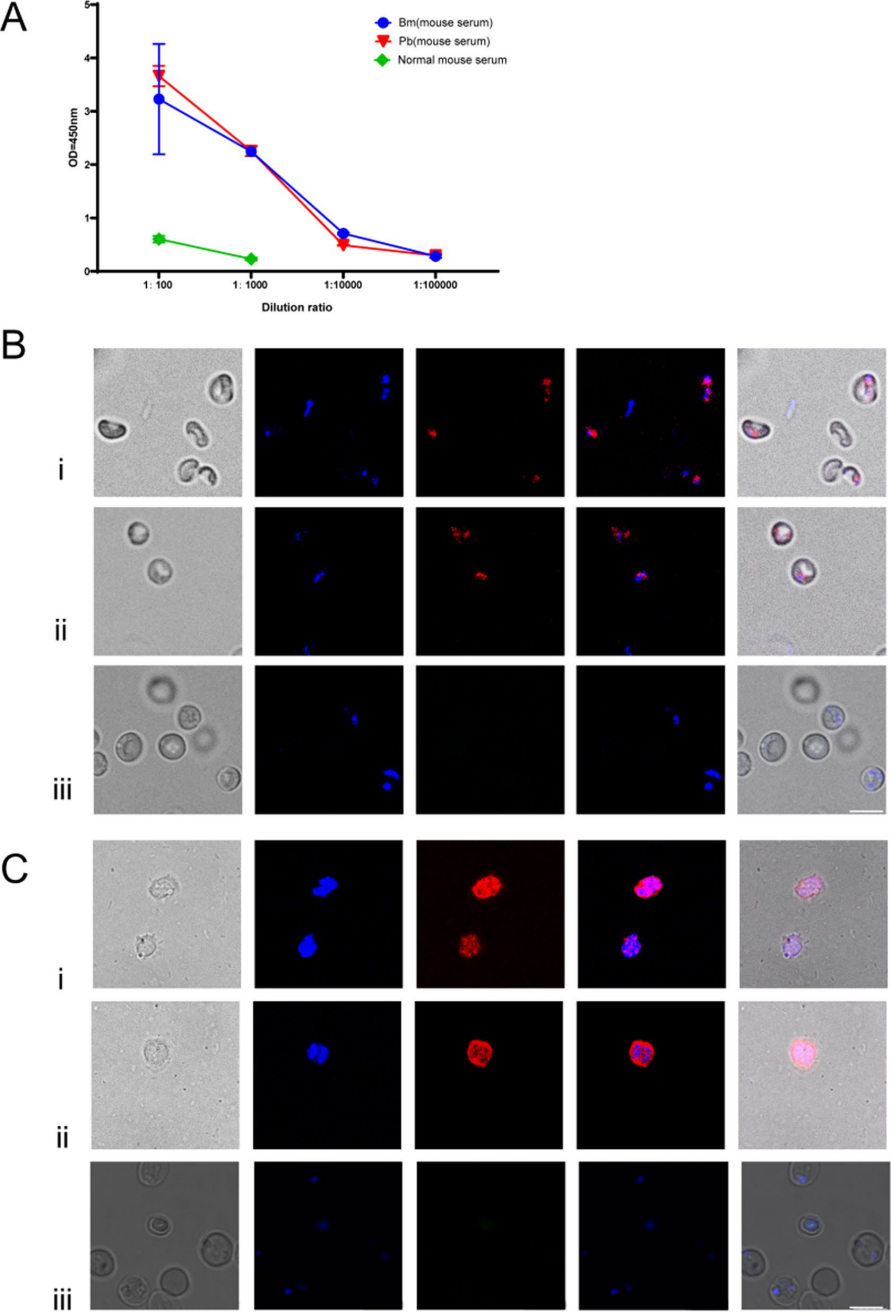
Antigenicity of r*Bm*8 and its subcellular localization. **a** Evaluation of antigenicity by ELISA with pooled sera from 5 mice infected with *B. microti* or *P. berghei*, respectively. **b** Localization of *Bm*8 in iRBCs of *B. microti* detected by IFA:* i*,* ii* iRBCs were co-incubated with anti r*Bm*8 sera;* iii* co-incubation of iRBCs infected with *B. microti*, with pooled sera of normal mice as the control groups. **c** Localization of *Bm*8 in iRBCs of *P. berghei* detected by IFA:* i*,* ii* iRBCs were co-incubated with anti-*rBm*8 sera;* iii* co-incubation of iRBCs infected with *P. berghei* with pooled sera of normal mice as the control groups. Scale bars: 5 μm. iRBCs, red blood cells infected with parasites; IFA, immunofluorescent assay; rBm8, recombinant *B. microti* protein 8

To validate the antigenicity of r*Bm*8, we used an indirect ELISA to detect r*Bm*8-specific antibodies from the sera of *B. microti*- and *P. berghei-*infected mice. The results suggested that r*Bm*8 could be recognized by the sera collected from both *B. microti*- and *P. berghei*-infected mice (Fig. 2a). Alternatively, both *B. microti* and *P. berghei* could be detected by anti-r*Bm*8 sera in the RBCS infected by *B. microti* and *P. berghei*, respectively. These results suggested that *Bm*8 and its *Plasmodium* homolog are localized in the cytoplasm of *B. microti* and *P. berghei.* As negative controls, iRBCs from mice infected with *B. microti* or *P. berghei* did not react with pooled sera of normal mice in the control groups (Fig. 2b, c).

### Active immunization with *Bm*8 partially protects mice against *B. microti* or* P. berghei* infection

**Fig. 3:**
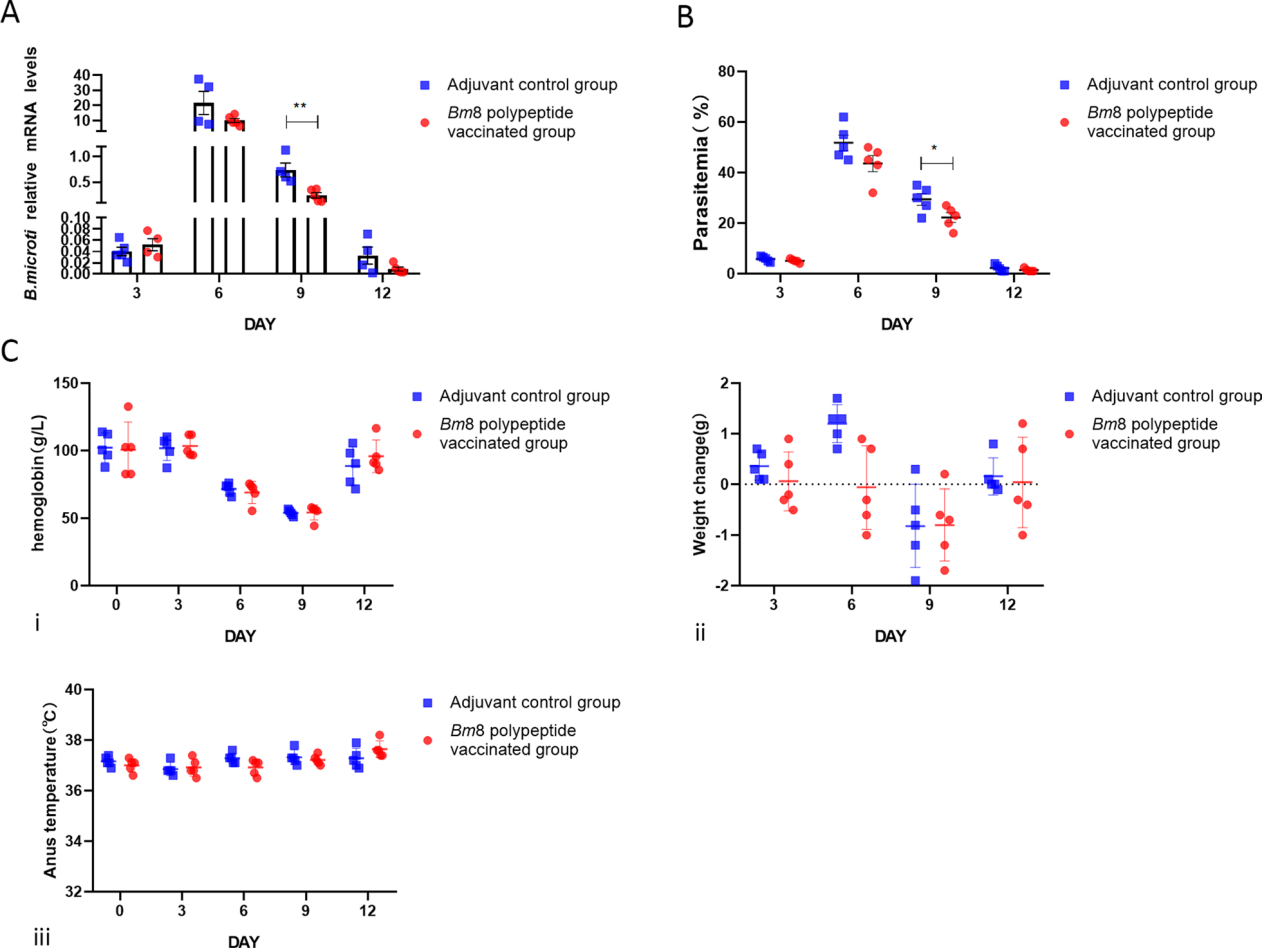
Active immunization protects against *B. microti* infection in BALB/c mice induced by the *Bm*8 polypeptide. **a** Detection of the copy number of the *B. microti* gene in BALB/c mice on days 3, 6, 9 and 12 post-infection by qRT-PCR. Asterisks indicate significant difference at **P < 0.01 vs the adjuvant group (*t*-test).** b** Parasitemia comparison in BALB/c mice infected with *B. microti* on days 3, 6, 9 and 12 post-infection detected by microscopy. Asterisk indicates a significant difference at *P < 0.05 vs the adjuvant group (*t*-test). **c** Severity of babesiosis in mice receiving active immunization with *Bm*8 polypeptide versus the control group, on days 3, 6, 9 and 12 post-infection:* i*–*iii* changes in Hb level (*i*), body weight (*ii*) and body temperature (*iii*). Bm8, *B. microti* protein 8; Hb, hemoglobin; mRNA, messenger RNA; qRT-PCR, real-time quantitative PCR

In this part of our study, we assessed whether active immunization of mice with *Bm*8 polypeptide affected *Babesia* infection. After active immunization, a high level of *Bm*8 antibody was detected in the sera of mice (Additional file 4: Figure S3 A, B). Compared with the control group, the copy numbers of the *Babesia* gene and parasitemia level of the immunized mice on day 9 post-infection were reduced (Fig. 3a, b). In addition, the changes in Hb level, body weight and anal temperature, which reflect the severity of babesiosis in BALB/c mice, were comparable between the immunized and the control group. Although the parasitemia in the immune group was lower than that in the control group, there was no significant difference in the indicators of disease severity, such as hemoglobin concentration, body weight and temperature (Fig. 3c, i, ii, iii).

**Fig. 4:**
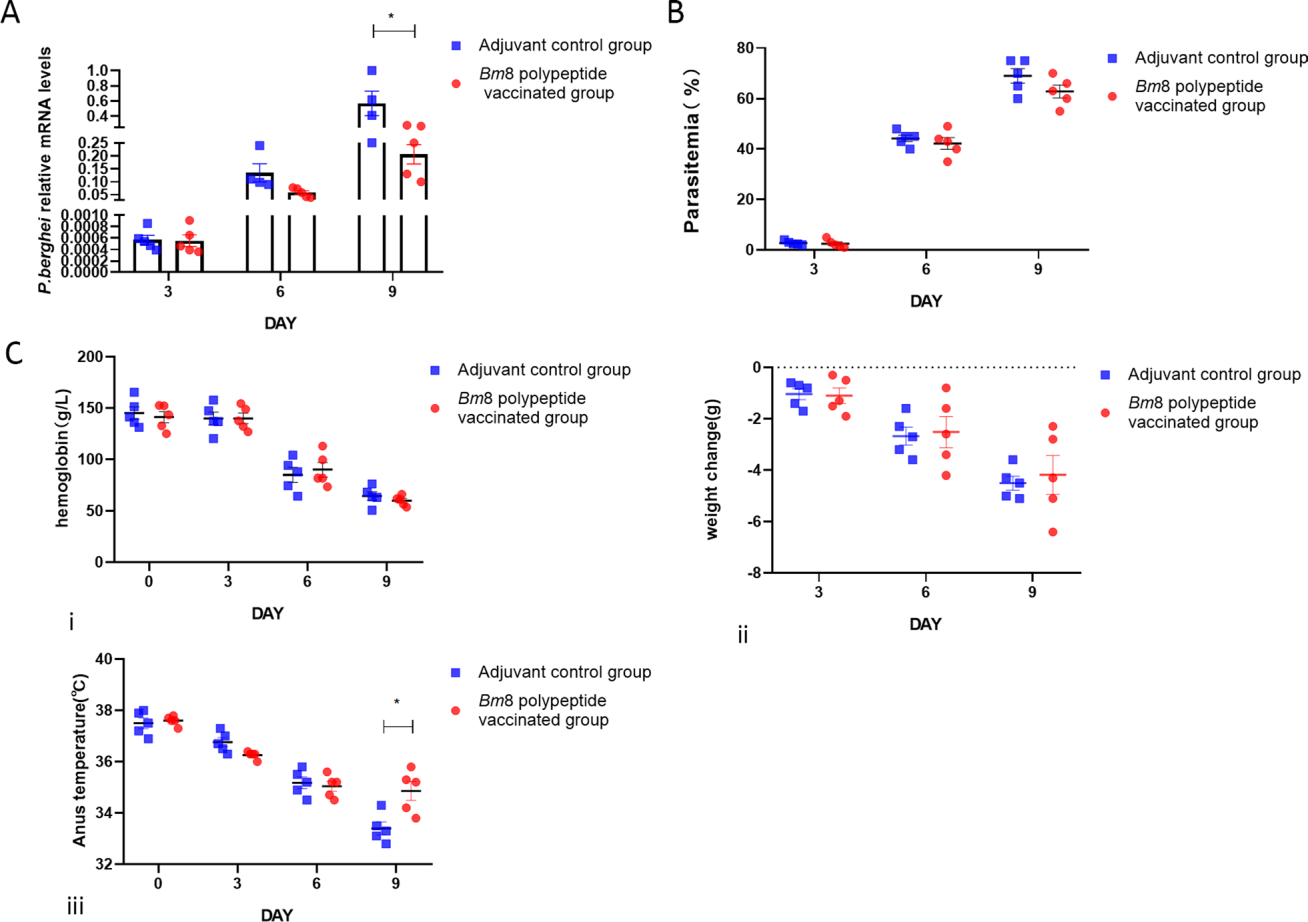
Comparison of body indicators between immunized mice and control mice infected with *P. berghei* after active immunization with *Bm*8 polypeptide or adjuvant, respectively. **a** Detection of copy number of *P. berghei* gene in BALB/c mice on days 3, 6, 9 and 12 post infection by qRT-PCR. Asterisk indicates a significant difference at *P < 0.05 vs the adjuvant group (*t*-test). **b** Parasitemia comparison in BALB/c mice infected with *P. berghei* versus control mice on days 3, 6, 9 and 12 post infection detected by microscopy. **c** Severity of babesiosis in mice receiving active immunization with *Bm*8 polypeptide versus the control group:* i*–*iii* changes in Hb level (*i*), body weight (*ii*) and body temperature (*iii*) in BALB/c mice challenge infection with *P. berghei* on days 3, 6 and 9 post infection. Asterisk indicates a significant difference at *P < 0.05 vs the adjuvant group (*t*-test). Bm8, *B. microti* protein 8; Hb, hemoglobin; mRNA, messenger RNA; qRT-PCR, real-time quantitative PCR

Next, we evaluated whether the active immunization of mice with *Bm*8 polypeptide affects *Plasmodium* infection. The copy number of the *Plasmodium* 18S RNA gene on day 9 day post infection were lower in the immunized mice than in the control group (Fig. 4a, b). The anal temperature was higher in mice in the immunized group than in the control group on day 9 post *P. berghei* infection (Fig. 4 c, iii). There was no significant difference in Hb concentration and body weight (Fig. 4c, i, ii).

### Passively immunization with *Bm*8 antisera partially protects mice against *B. microti* or* P. berghei* infection

**Fig. 5:**
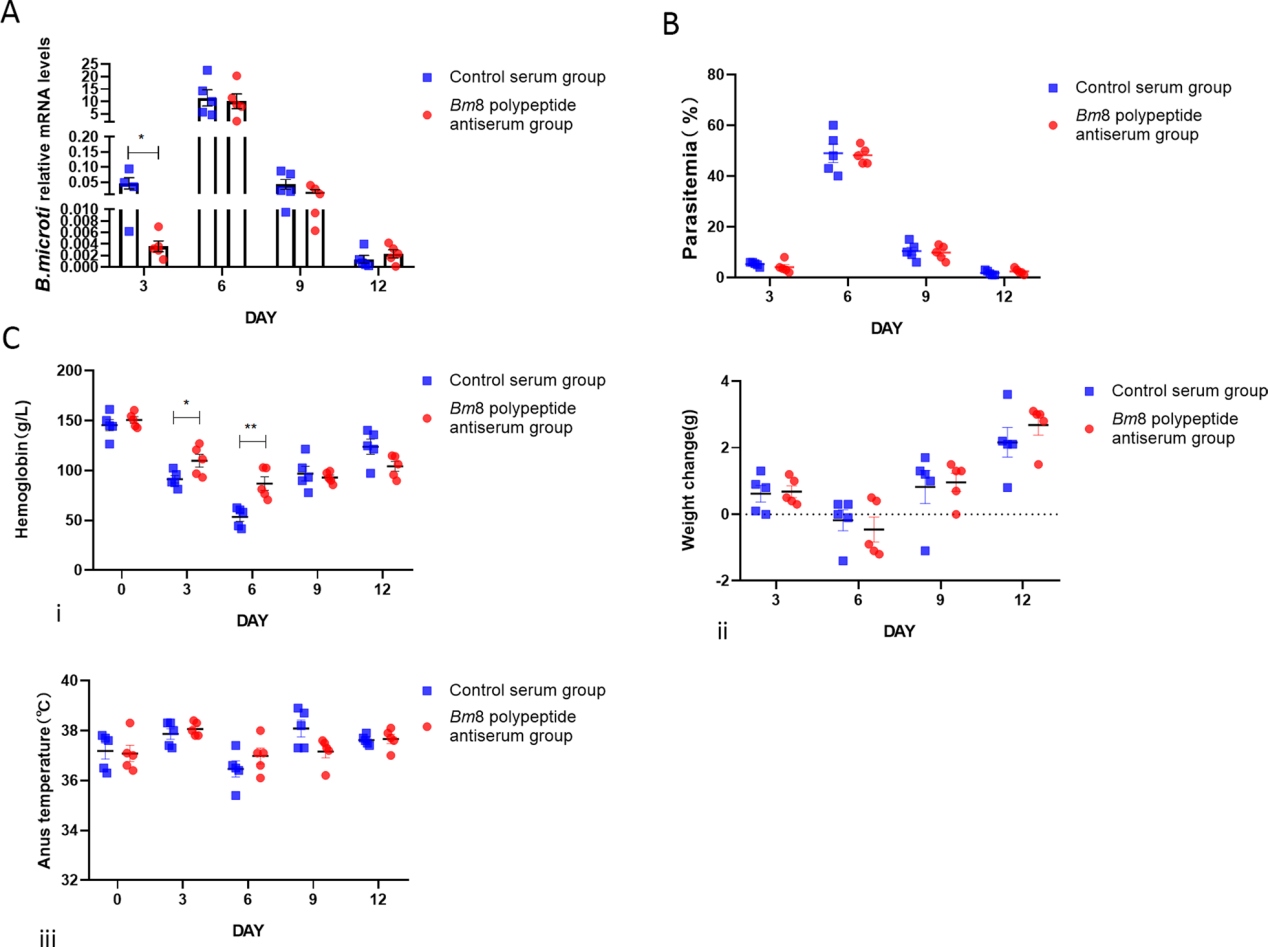
Passive immunization protects against *B. microti* infection in BALB/c mice induced by *Bm*8 antisera. **a** Detection of the copy number of *B. microti* gene in BALB/c mice on days 3, 6, 9 and 12 post infection by qRT-PCR. Asterisk indicates significance at *P ＜ 0.05 vs control serum group (t-test). **b** Parasitemia comparison in BALB/c mice infected with *B. microti* on days 3, 6, 9 and 12 post infection versus control group detected by microscopy. **c** Severity of mice babesiosis in mice receiving passive immunization with *Bm*8 polypeptides antisera versus those receiving control sera:* i*–*iii* changes in Hb level (*i*), body weight (*ii*) and body temperature (*iii*) in BALB/c mice challenge infection with *B. microti* on days 3, 6, 9 and 12 post infection. Asterisks indicate a significant difference vs control serum group at *P < 0.05 and **P < 0.01 (t-test). Bm8, *B. microti* protein 8; Hb, hemoglobin; qRT-PCR, real-time quantitative PCR; mRNA, messenger RNA; real-time quantitative PCR

**Fig. 6:**
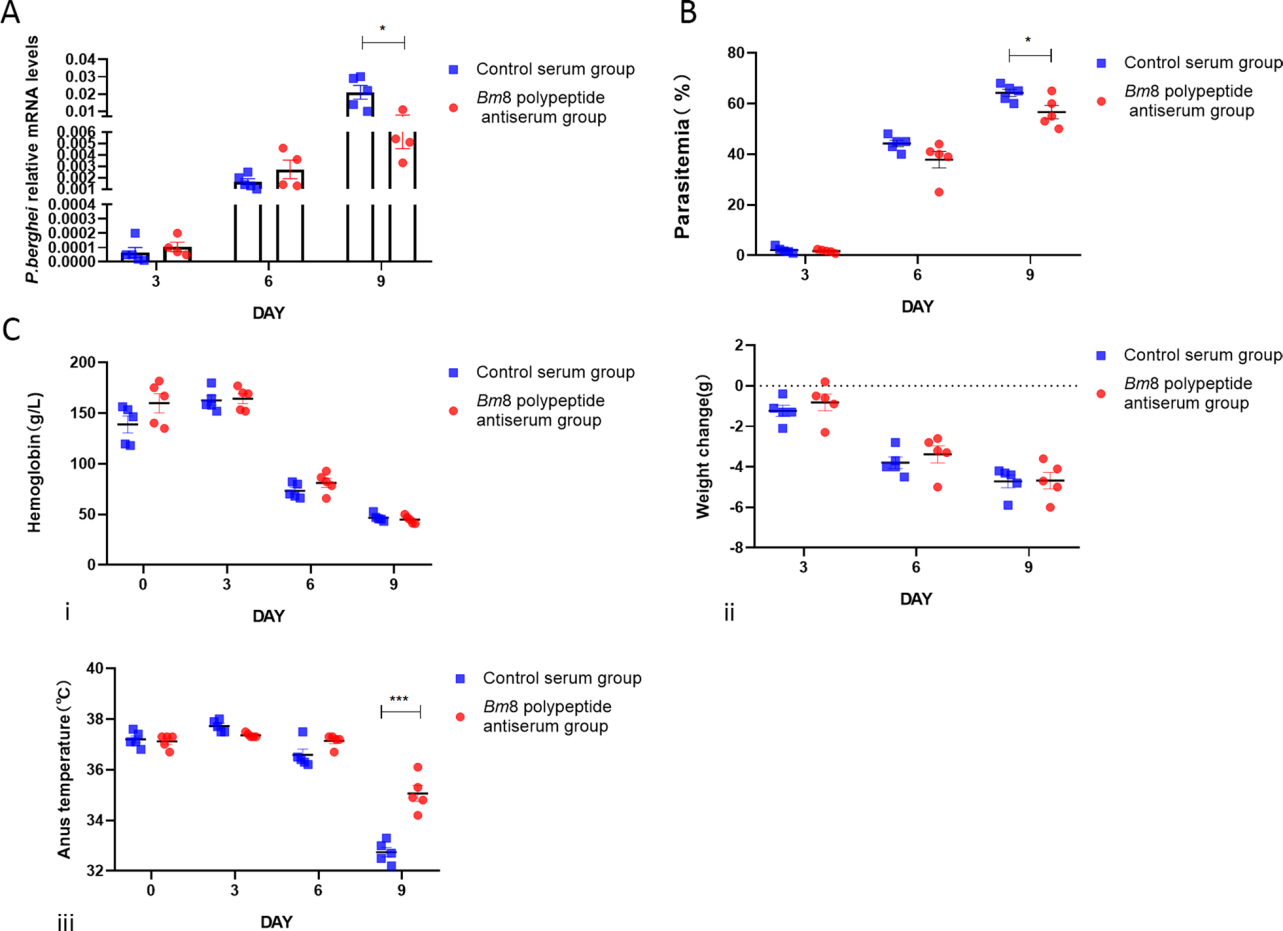
Passive immunization protects against *P. berghei* infection in BALB/c mice induced by *Bm*8 antisera. **a** Detection of the copy number of *P. berghei* gene in BALB/c mice on days 3, 6, 9 and 12 post infection by qRT-PCR. Asterisk indicates significant difference at *P < 0.05 vs adjuvant group (*t*-test). **b** Parasitemia comparison in BALB/c mice infected with *P. berghei* on days 3, 6 and 9 post infection versus control group detected by microscopy. Asterisk indicates significant difference at *P < 0.05 vs adjuvant group (*t*-test). **c** Severity of mice babesiosis in mice passive immunization with *Bm*8 polypeptides antisera group verus those receiving control sera:* i*,* ii*,* iii* changes in Hb level (*i*), body weight (ii) and body temperature (iii) in BALB/c mice challenge infection with *P. berghei* days 3, 6 and 9 post infection.** a**,** b**,** c** (*i*). Asterisks indicate a significant difference at ***P < 0.001 (t-test). Bm8, *B. microti* protein 8; Hb, hemoglobin; qRT-PCR, real-time quantitative PCR; mRNA, messenger RNA; real-time quantitative PCR

To investigate the protective effect of *Bm*8 antiserum against* B. microti* and* P. berghei* infection, the mice were injected with Bm8 antiserum 24 h before being challenged with the parasites. A booster immunization was given 4 days after the infection. We found that the copy number of the *Babesia* gene on day 3 post infection was lower in mice infected with *B. microti* than in the mice of the control group. However, there was no significant difference in body weight between the immunized group and the control group (Fig. 5a, b). The Hb concentration on days 3 and 6 post infection was higher in the immune group compared to the control group (Fig. 5c, i); there was no significant difference in body weight and temperature between the immune group and the control group (Fig. 5c, ii, iii). Following *P. berghei* infection, the copy number of the *Plasmodium* gene and parasitemia of the immunized mice on day 9 post infection were lower than those in the control group. Again, there was no significant difference in body weight between the immunized group and the control group (Fig. 6a, b). The anal temperature of the mice in the immunized group was slightly higher than that of the control group after 9 days of infection with *P. berghei.* (Fig. 6c, iii); there was no significant difference in Hb concentration and body weight between the immune group and the control group (Fig. 6c, i, ii).

## Discussion

Vector-borne blood parasites are responsible for some of the most widespread, serious and poorly controlled diseases globally, including malaria caused by *Plasmodium* and babesiosis caused by *Babesia* spp. There is an urgent need to better understand the mechanisms of transmission and the invasion process of these parasites to the host cells in order to optimize control methods, including the development of vaccines [26]. In this study, we cloned and expressed a novel highly conserved membrane-associated antigen of *B. microti*, known as *Bm*8, and performed preliminary functional research by synthesizing one of its peptide fragments. Based on structural prediction and antigenicity analysis, we found that the protein *Bm*8 had high homology in the Apicomplexan protozoa, including *P. berghei*, *P. vivax*, *P. falciparum, B. bovis* and *T. annulata*. In addition, members of the *Babesia* and *Plasmodium* belonging to the same apicomplexan hemoprotozoa undergo a complex life-cycle involving vectors and mammalian hosts and have some similar subcellular structures. These two parasitic protozoa can secrete multiple merozoite apical membrane and organelle-related proteins that may play important roles in the process of these protozoa invading host cells [27, 28].

Interestingly, a series of published studies identified pepsin-family aspartyl proteases (APs) of *B. microti* to have a phylogenetic relation to the homologs of *P. falciparum* plasmepsins (PfPM I–X) and *T. gondii* aspartyl proteases (TgASP1–7). Based on these analogies with plasmodial plasmepsins, the APs were believed to represent valuable targets for the development of cross-species drugs against infection by apicomplexan protozoa [29–31]. Also, a series of cases of mixed infection of *Babesia* and *Plasmodium* in febrile patients in malaria-endemic areas located on the border area between China and Myanmar have been reported over the years [32]. Other cases of mixed infection have been reported in the Kilosa region in Tanzania, Africa, Guinea in West Africa and other malaria endemic areas [33–35]. It is widely thought that when *Babesia* and *Plasmodium* are co-infecting one host, they may interact with each other and compete to invade the host cells [36, 37]. The authors of one study reported that a number of rhesus monkeys imported from China had a low parasitemia of *Plasmodium* after being challenged with infections. Further analysis revealed that these rhesus monkeys from Guangxi, China had previously been infected with *B. microti*, suggesting that these monkeys previously infected with *Babesia* may be less sensitive to malaria or that the progress of malaria after infection is slower than that in control animals [36]. The existence of co-infections with vector-borne protozoa has prompted us to consider whether a vaccine can be designed to address multiple similar protozoa infections. Given the interactions between *Babesia* and *Plasmodium* parasites and the observation that erythrocyte molecules interact with each other, the ligands formed may play a role in the parasite interface to facilitate successful invasion [10, 37]. Several conserved sequences among the apicomplexan protozoa and their related invasion and conservation mechanisms suggest that these targets may induce cross-immune responses during co-infection by *Babesia* and *Plasmodium* [38, 39].

Although a conserved region with high antigenicity in *Bm*8 was identified through sequence comparison, there have been few related studies on these proteins containing the same conservative sequence. In the present study, we sought to investigate the potential protective effect of the* Bm*8 polypeptide in a murine model infected with either *B. microti* or *P. berghei*. Based on the results of active immunization, the protective effect of *Bm*8 polypeptide was more evident during the middle and late stages of both *B. microti* and *P. berghei* infections, particularly on the day 9 post infection. However, the immune protective effect induced by this target antigen was relatively weak, which may be related to the higher concentration of the parasites during the challenge infection and the lower concentration of the antisera utilized in our experiment. In the study of active immunization in mice infected with *P. berghei* and the control group, the results of qPCR showed that on day 9 post infection, the relative copy number of peripheral blood parasites in the immunized group was lower than that in the control group. Similarly, in the study of passive immunization with *Bm*8 antisera in *B. microti*-infected group and control group, the results of qPCR showed that on day 3 post infection, the relative copy number of the parasites in the peripheral blood of the immunized mice was lower than that in the peripheral blood of the control group. These results showing statistically significant differences by qPCR are not completely consistent with the results of peripheral blood smears during the same period. One reason may be that qPCR is a more sensitive method for detecting worm concentrations that are biologically active or in their parasitic life-cycle (e.g. parasites that are invading erythrocytes), whereas the detection of parasitemia by blood smears is more intuitive and concrete, but relatively lagging behind the infection. Another reason for the inconsistent results between the qPCR and blood smear may be due to the weak immune protection induced by the target peptides and its anti-sera, such that the difference between the immune group and the control group was not significantly sufficient. Another issue that cannot be ignored is that the cloning and expression efficiency of the target protein *Bm*8 was low, resulting in insufficient protein in the immune protection experiments. Therefore, further optimization of experimental methods is required to better assess the function of the conservative sequence, and the efficacy of *Bm*8 as a protective antigen needs to be verified through in vivo and in vitro experiments.

Hb concentration, body weight and body temperature are indicators that reflect the severity of the disease, and they may be distinctly different when obvious protections are induced in the immune group [23]. In this study, we found that Hb concentration was significantly higher in the immune group than in the control group at days 3 and 6 after passive immunity regarding Bm infection. This result is consistent with the obvious reduction in the parasite level and suggest that passive immunity of anti-*Bm*8 serum-induced protection against* Babesia* infections.

In the control group of *P. berghei* infections, the results shown in Figs. 4c and 6c show a comparison of mouse body temperature. We found that the body temperature of the immunized group was higher than that of the control group and closer to that of normal mice. We speculate that the reason for this is that about 10 days after infection with *P. berghei*, the infected mice no longer had fever but they did show a significant reduction in RBCs, presenting with severe anemia and a gradually decreasing body temperature which may be due to extreme physical weakness. The body temperature was closer to that of the normal group, possibly indicating that the severity of the disease is less than that in the control group.

There are a number of limitations to this study. First, although IFA is a commonly used method for labeling proteins with fluorescent antibodies for localization [39, 40], we were unable to clearly localize the target protein,* Bm*8 in this study. It was predicted that there are two transmembrane domains in this protein, but the IFA did not show the membrane localization, and this aspect requires further evaluation. Meanwhile, we speculate that *Babesia* spp. are unicellular eukaryotes with organelles. Therefore, the proteins localized on the membrane may also be proteins on the plasma membrane of the organelle. If further experiments verify that* Bm*8 is a protein in the cytoplasm, then mRNA vaccines may be promising vaccines. mRNA can be introduced directly into animal body cells (vaccine injected into humans) by the special delivery of gene fragments (DNA or RNA) encoding* Bm*8 protein, producing antigenic protein through the protein synthesis system of host cells to induce an immune response to that antigenic protein for the purpose of disease prevention and treatment [41]. It has also been shown in the literature that erythrocytes themselves can act as carriers for systems that deliver substances such as drugs, enzymes, peptides and antigens in vivo; thus, if it is clear that the parasite is localized in the cytoplasm after invasion of erythrocytes, this mechanism has implications for the development of intracytoplasmic vaccines [42]. Secondly, it has been reported that self-elimination of* Babesia* protozoa is possible, especially in cases of low parasitemia. This phenomenon has also been described in infections caused by a number of *Babesia* species [43]. Clinically, *B. microti* infection in humans in the presence of other underlying diseases can be fatal, while most of the infections are relatively not severe [4, 44]. This immune clearance mechanism will inevitably function as interference in our protection evaluation experiment. We compared the infection of the immune group and the control group using the same infection dose at the same time. Third, due to the low expression of* Bm*8 protein, we synthesized the conserved antigenic peptides to conduct the immune protection experiments. The protective studies in this research used the synthesized peptides, rather than the* Bm*8 protein itself. Meanwhile, if we can identify some key conserved antigenic peptides and design vaccines through synthesized peptides, we will have developed an effective way to overcome the complexity of protein expression and purification processes, as well as yield uncertainty. Finally, further research is needed on the interaction between the target protein* Bm*8 and the erythrocyte membrane receptors.

## Conclusion

Our study identified a conserved erythrocytic membrane-associated protein from *B. microti*, named* Bm*8. The results of our study suggest that *Bm*8 could be a promising subunit vaccine candidate targeting the blood stage of hemoprotozoa, including babesiosis and *Plasmodium* infections transmitted by vectors, based on the observed protection in both active and passive immunization studies against *B. microti* and *P. berghei* infections in mice. Moreover, since *P. berghei* is used as a model for human malaria, our findings could have implications for the development of effective malaria vaccines. Overall, this study provides new insights into preventing and controlling intraerythrocytic protozoa infections transmitted by vectors.

### Supplementary Information

**Additional file 1. Word file S1:** Amino acid sequence of target protein *Bm*8 (noted as “conserved *Plasmodium* protein, unknown function” online).

**Additional file 2. Figure S1:** Expression and purification of r*Bm*8.** A** Purification of r*Bm*8 confirmed by SDS-PAGE electrophoresis, by loading with r*Bm*8 without GST tag about a MW of approximately 57 KDa. **B** Western Blot assay of the purified rBm8:* a* probed with normal mouse serum,* b* probed with anti-*B. microti* mice serum. The red arrow indicates the position of the target protein* Bm*8 band.

**Additional file 3.** **Figure S2:** Prediction of linear epitope of *Bm*8 antigen and synthesis of target peptide (CYDPEKSNSAEW).** A** Antigenic analysis of *Bm*8, with five main antigens predicted.** B** Synthesis report of target peptide.** C** Preparation of rabbit antiserum of target peptide.

**Additional file 4.** **Figure S3:** Evaluation of active immunity affection. In each group, 5 mice were used to set up the active immunization models. The control group was immunized with equivalent adjuvant.** A** Determination of active immune antibody titer of *Bm*8 polypeptide (before challenge infection with *B. microti*).** B** Determination of active immune antibody titer of *Bm*8 polypeptide (before challenge infection with *P. berghei*).

## Data Availability

All data are included as figures and Supplementary Information in the article.
