# Supplementary material for: A conserved protein of Babesia microti elicits partial protection against Babesia and Plasmodium infection
Source: Parasit Vectors. 2023 Aug 30;16:306. doi: 10.1186/s13071-023-05825-x (PMC10469411; doi:10.1186/s13071-023-05825-x)
Supplement: Supplementary file 1 — Additional file 1. Word file S1: Amino acid sequence of target protein Bm8 (noted as “conserved Plasmodium protein, unknown function” online). [file 13071_2023_5825_MOESM1_ESM.docx]

“XP_021338580”：

MYAPSGATLALTLFAVLNIYFTFSYGKSSQSSTTIAIPVYLSEGGLTISVSVASKTHQLALSGRLEGIYLFSNKIPNCQNCYDPEKSNSAEWCNNPNEKCNPIISKFVCNRGKKLDYNWAVKTGPFAFDSLYYVPQNIQGYEQIQIESHAVSGSSFFNTNFNAPVKRTFLGTIPIALIASINSYPNWPLFKNISGIFGLVGPSLCCRESSIWWSIIGEYNNTFQIDIDGDFSQPKDFEQVGNLVLGASEDEFVWSNPRHIGGLYSDALMQFTVYNFKMCNVELFGQTSSNWEAIIDPTCRCLGLPKNFWLSLMKHLPVDPNDCLNDENLPRLCKLKSPFPKLPIISFTLKDDPRSPKLEIPLQSLLFKLNDNDTEQRLCIVPTDISGVNRSKAITTFPSIKFGYQVLRNFKVAVDQGNCKIGFLNRGKFIGSDEVCSAKVTCKGDQVYCAAMNTCLNPECSIWLLKSYDPNSGTCKFNMWAMVFLWITIALIGLIDIHSYLSYRRLLTHAKRLCK
