# Supplementary figures and images for: A conserved protein of Babesia microti elicits partial protection against Babesia and Plasmodium infection
Source: Parasit Vectors. 2023 Aug 30;16:306. doi: 10.1186/s13071-023-05825-x (PMC10469411; doi:10.1186/s13071-023-05825-x)

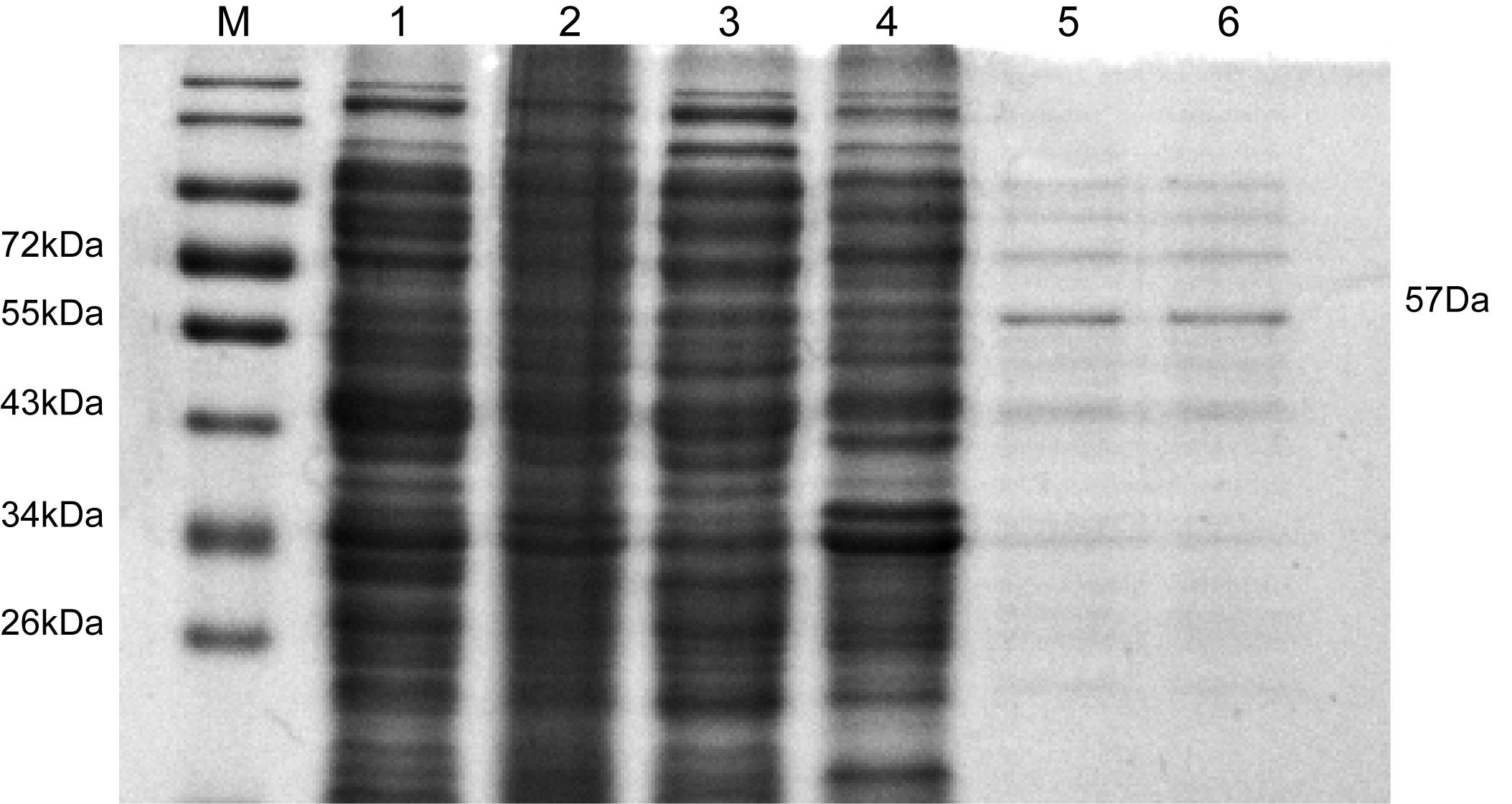

Supplement: Supplementary file 2 — Additional file 2. Figure S1: Expression and purification of rBm8. A Purification of rBm8 confirmed by SDS-PAGE electrophoresis, by loading with rBm8 without GST tag about a MW of approximately 57 KDa. B Western Blot assay of the purified rBm8: a probed with normal mouse serum, b probed with anti-B. microti mice serum. The red arrow indicates the position of the target protein Bm8 band. [file 13071_2023_5825_MOESM2_ESM.tif]

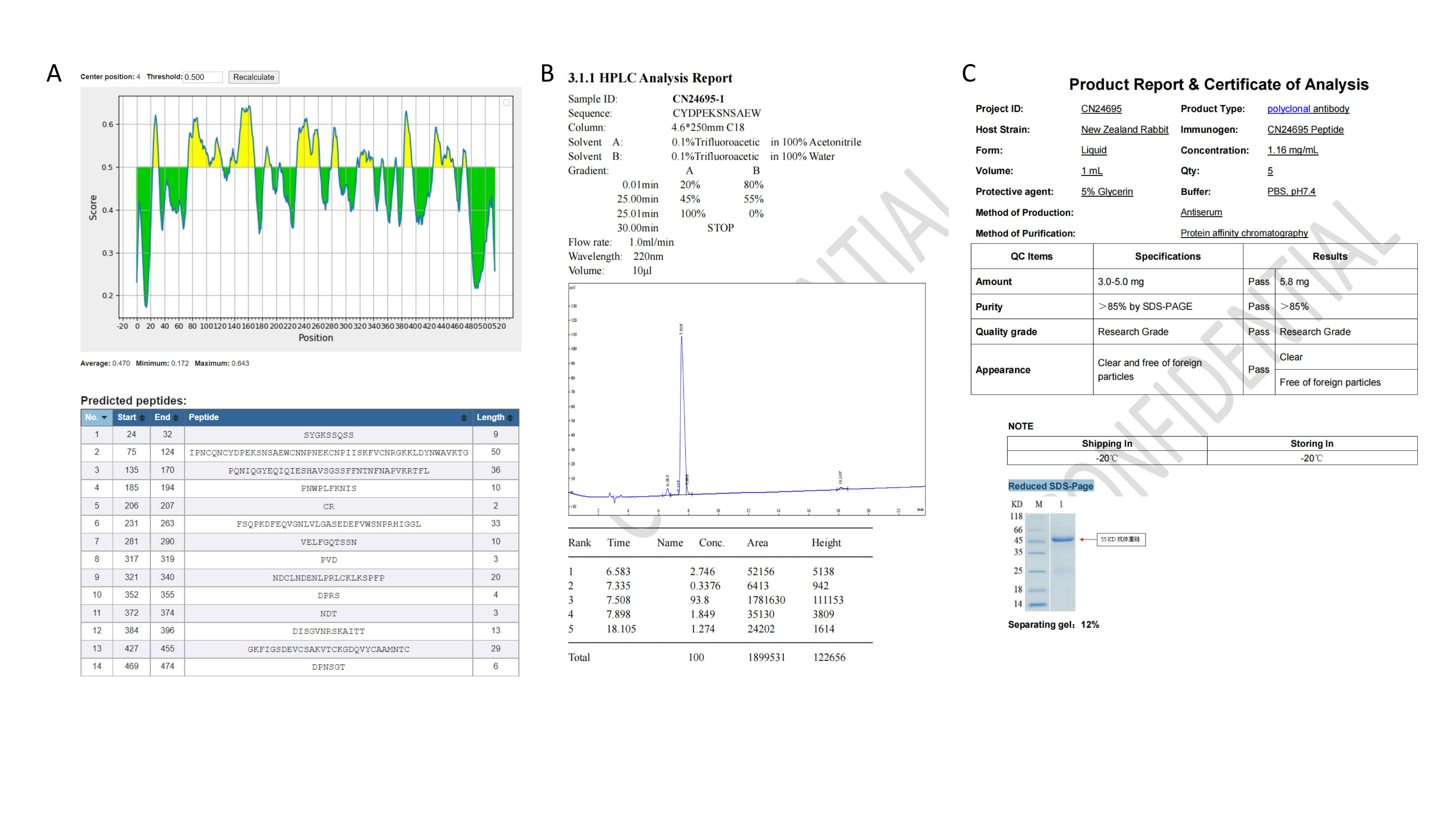

Supplement: Supplementary file 3 — Additional file 3. Figure S2: Prediction of linear epitope of Bm8 antigen and synthesis of target peptide (CYDPEKSNSAEW). A Antigenic analysis of Bm8, with five main antigens predicted. B Synthesis report of target peptide. C Preparation of rabbit antiserum of target peptide. [file 13071_2023_5825_MOESM3_ESM.tif]

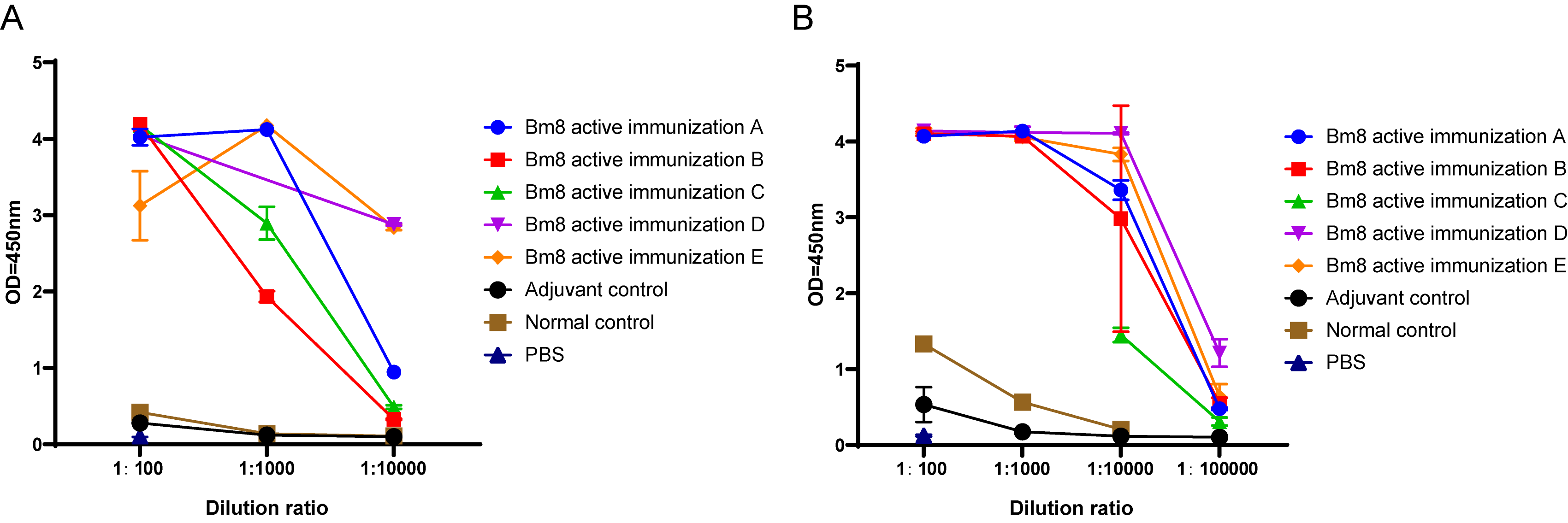

Supplement: Supplementary file 4 — Additional file 4. Figure S3: Evaluation of active immunity affection. In each group, 5 mice were used to set up the active immunization models. The control group was immunized with equivalent adjuvant. A Determination of active immune antibody titer of Bm8 polypeptide (before challenge infection with B. microti). B Determination of active immune antibody titer of Bm8 polypeptide (before challenge infection with P. berghei). [file 13071_2023_5825_MOESM4_ESM.tif]
